# Oguchi Disease Associated with Keratoconus

**DOI:** 10.18502/jovr.v16i1.8262

**Published:** 2021-01-20

**Authors:** Ahmad Mirshahi, Narges Hassanpoor, Hassan Khojasteh, Mohammad Reza Baradaran, Hooshang Faghihi, Alireza Lashay

**Affiliations:** ^1^Retina & Vitreous Service, Farabi Eye Hospital, Tehran University of Medical Sciences, Tehran, Iran; ^2^Vanak Eye Clinic, Tehran, Iran

##  PRESENTATION

**Figure 1:**
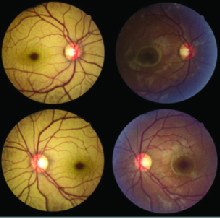
Mizuo-Nakamura phenomenon. Fundus photo of right and left eyes before (left column) and after (right column) 6 hr of overnight dark adaptation. All photos have been taken in normal illumination. Abnormal yellow sheen disappeared after dark adaptation.

**Figure 2:**
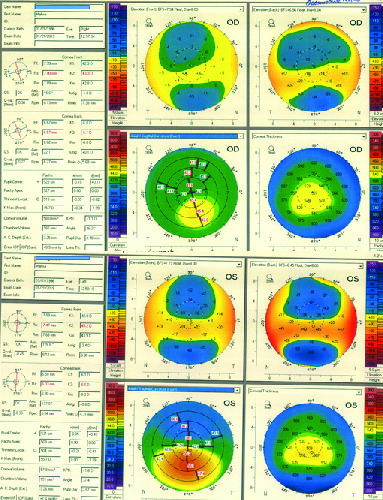
Pentacam of right (upper row) and left (lower row) eyes. Right eye Pentacam shows inferior steepening, high I-S value, posterior elevation, and inferior displacement of the thinnest point. Pentacam of left eye shows significant inferior steepening, increased keratometries, anterior and posterior elevation, and inferior displacement of the thinnest point that is compatible with keratoconus diagnosis.

A 22-year-old female came to a cornea specialist in our center to do refractive surgery. The best-corrected visual acuity was 20/20 in both of her eyes with the following refraction: OD: –4.5–0.75 x 180 and OS: –4.75–2.00 x 110. Scissors motion was obvious in her left eye during refraction. In funduscopic evaluation, an abnormal yellow to brown sheen was obvious in her both eyes (Figure 1, right column). Other ocular examinations were within normal limits and patient had no history of any other systemic or ocular disease. Drug history and family history of ocular diseases were negative. Due to scissors motion and abnormal Pentacam (Figure 2), she has been diagnosed with keratoconus, her refractive surgery has been held, and corneal cross linking (CXL) was suggested to her. Both eyes optical coherence tomography (OCT) were completely normal but due to abnormal yellow sheen in her both eyes funduscopy, she was referred for further evaluation to us before CXL. She denied any night blindness or decreased vision in her both eyes. Oguchi disease diagnosis was made with presence of obvious Mizuo-Nakamura phenomenon (Figure 1) and was confirmed with genetic testing. Her electroretinography (ERG) was done based on the International Society for Clinical Electrophysiology of Vision (ISCEV) protocol (Metrovision, Pérenchies, France). Due to rapid loss of dark adaptation by a short light exposure, dark adapted fundus photo and ERG have been done in different visits but with same instruments. Fundus photos have been captured by Canon CR-2 AF Retinal Camera. There was not any abnormality in her both eyes OCT angiography (OCTA) by Optovue OCTA (Fremont, CA, USA).

**Figure 3:**
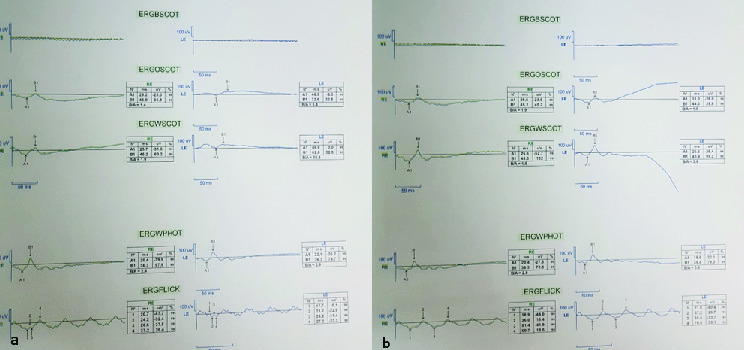
ERG before (a) and after (b) 6 hr of dark adaptation. Her first ERG (a) was performed after 30 min of dark adaptation which showed a severely reduced b-wave amplitude with a mild reduction of the a-wave that improved after 6 hr of overnight dark adaptation (b).

Genetic testing has shown a homozygous mutation in SAG (NM_000541.5) gene, variant c.874C>Tp.R292 which is compatible with type one Oguchi disease.

##  DISCUSSION 

Oguchi disease is a type of congenital stationary night blindness (CSNB) with autosomal recessive inheritance. Patients usually have normal visual acuity and do not complain from night blindness. The disease is very rare and around 50 cases have been reported up till now. Most of the cases are from Japan and Pakistan.^[1]^ Patients have an abnormal fundus color that is described as having yellow to brown sheen or metallic appearance. Prolonged dark adaptation can recover rhodopsin and re-normalize fundus color (Mizuo-Nakamura phenomenon).^[2,3,4]^

Oguchi disease has been reported in association with retinitis pigmentosa^[4]^ and diabetic retinopathy,^[1]^ but there is no report of its association with keratoconus or any other corneal abnormality up till now. However, there was a report of X-linked CSNB associated with keratoconus in 2006 from UK by Nguyen *et al*.^[5]^

Here, we report the first case of association of this disease with keratoconus in the world. To the best of our knowledge, this is the second case of Oguchi reported from Iran^[1]^ but the first genetically proven Oguchi disease type 1 of Iran and Middle East.

The other case from Iran has shown negative ERG in photopic state with near flat ERG in scotopic condition.^[1]^ Another case of Oguchi disease was reported by Francois *et al* with absent scotopic waves in 1956.^[1]^

In conclusion, Oguchi disease can be seen with keratoconus. Although it could be accidental due to high prevalence of keratoconus in our population, further reports in future may suggest a pathogenic linkage between them.

##  Financial Support and Sponsorship

Nil.

##  Conflicts of Interest 

There are no conflicts of interest.
